# Recent Experiences with Puerperal Septicæmia

**Published:** 1892-06

**Authors:** W. D. Haggard

**Affiliations:** Professor of Gynecology in the Medical Department of the University of Tennessee, Nashville


### RECENT EXPERIENCES WITH PUERPERAL SEPTICÆMIA.

BY

W. D. HAGGARD, M. D.,

Professor of Gynecology in the Medical Department of the University of Tennessee, Nashville.

The following cases of puerperal septicæmia occurred in my practice recently:

CASE I.—Mrs. S., age thirty-nine, was delivered, on the 9th of June, of her seventh child by a midwife. On the 12th and 13th she had chilly sensations, followed by fever ; on the 14th a decided chill, at which time Dr. Jordan was called. He administered calomel, quinine and opium, which acted well. On the 15th I was called in consultation. We now recognized puerperal septicæmia, which had been very insidious in its approach. Her bowels had been moved by the mercurial, and she had an anxious, distressed countenance. Skin of a yellowish hue ; face swollen ; tongue heavily coated ; breathing oppressed. Bowels were tympanitic and tender over the uterus ; pulse 130, temperature 104. Lochia scant and very oppressive ; highly colored urine. Had slept but little in forty-eight hours. The uterus was flushed with very hot water and irrigated with bichloride of mercury. She was ordered one tablespoonful of whisky every hour, sulphate of quinine, five grains every four hours, and the irrigatio was repeated at a corresponding interval.

On the 16th Dr. Richard Douglass was added to the consultation. It was decided to curette, which was done, but failed to bring away any placental tissue or blood clots which we feared might have been retained. A 5 per cent. solution of creoline was substituted for the bichloride and used every four hours for the next week. Quinine was continued. Diet consisted of milk and eggs. Her secretions were looked after and bowels kept open. She convalesced very slowly, but finally recovered.

CASE II.—Mrs. W. I., para, aged twenty-six, was delivered with forceps, having been in labor thirty hours. There was nothing abnormal in either the labor or delivery except a general atonic condition. The womb did not contract well, but there was no hemorrhage. She showed marked debility from the first. At 6 P. M. her pulse was feeble and registered 130 ; temperature 101. For the next week her pulse and temperature vacillated greatly, the former ranging from 100 to 130, while the latter ranged from 100 to 104, both declining after each irrigation. Creoline replaced the bichloride. The second week marked some little improvement in her general condition, although the uterus was tender and imparted a boggy feeling. This inclined me to think that the condition was becoming localized in the walls of the uterus. I contemplated abdominal section, but was deterred by her apparent improvement. On the fourteenth day after confinement she had a severe chill, lasting two hours, after which phlegmasia dolens was marked. My friend, Dr. J. B. Stephens, was called in consultation immediately after the chill and kindly assisted me in the conduct of the case. She died on the seventeenth day, her condition never justifying an operation after the chill.

Where did this woman get puerperal septicæmia?

During the progress of the former case I had seldom had such a run of obstetric engagements, having attended cases on the 17th, 20th, 22d and on the 30th, respectively.

On the night of the 31st I delivered a lady at 12 P.M., and went immediately to the case just reported, which terminated at 4 A.M., February 1st. Was this case one of heterogenetic origin? I think not.

1. Because it developed too soon after delivery (within twelve hours).

2. It seems improbable that I should infect her without infecting the woman delivered four hours previously.

3. The same methods were employed in both cases.

4. None of the intermediate cases were infected.

Hence I concluded she had some localized focus of infection existing prior to delivery which culminated in an explosion at the parturient effort.

I consider autogenetic infection a myth, but the latter case simulates it very closely. The first case was in excellent health previous to delivery, while the second had albuminuria.

The more advanced thinkers believe that the disease depends upon the introduction from without of germs of any of the specific diseases which come under the generic term of micro-organisms.

Infection occurs generally during the act of parturition, and is carried into the genitalia by neglect of the aseptic *régime*. However introduced, the germs find in the lochial discharge the best possible medium for their multiplication and development. The great bacterial activity which speedily follows their introduction gives rise to putrefactive changes, which result in the formation of pathogenic alkaloids known as ptomaïnes and leucomaïnes, which are supposed to produce the toxic effect, the symptoms of which are observed at the bedside of our cases of puerperal septicæmia.

This class of cases are said to be heterogenetic in their origin. Micro-organisms often gain admission to the genital track after abortion or through gonorrhœal infection, which result in intra-pelvic inflammation and tubo-ovarian diseases that furnish the gynecologist with much of his pelvic surgery. Exceptionally the subjects of these milder forms of tubo-ovarian trouble conceive. Gestation progresses normally, labor sets in, handicapped by the presence of a focus of infective material, pocketed, walled in, by the products of previous inflammation. The parturient act results in rupture, or in the escape of pus reeking with bacteria, which may or may not enter the uterine cavity. But whether it gains access to the womb or not, the peculiar blood-state of the woman, the wonderful activities of her entire organism, the great susceptibilities of her nature to the influence of septic infection at this juncture, render her system doubly liable to the invasion of the disease-producing germs.

Practically, it matters not through what avenue infection comes, it finds the puerperal woman in a peculiarly receptive condition. Cases complicated by the presence of pus, either in the tubes or ovaries, which existed prior to the onset of labor, and rupture during the parturient effort, constitute the nearest approach we have to auto-infection.

My rule of action is, in all cases requiring the introduction of the hands or instruments into the cavity of the uterus, to use an injection of mercuric bichloride, one to three thousand.

When labor is perfectly normal I leave the uterus to take care of itself. I visit my cases every day for five days after delivery. If no evidences of septic trouble manifest themselves by that time I consider my patient safe. If, however, she shall have a rigor or slight chill, which usually occurs about the third or fourth day, with tenderness outlined by the uterus and broad ligament, oppressive lochia and a rise of temperature, I institute heroic measures at once.

The uterine cavity is douched with conveniently warm sterilized water, followed by sublimate irrigation. This is best accomplished by the introduction of a large glass tube or hard rubber nozzle attached to a fountain syringe. I ignore the generally accepted danger of using uterine irrigation except through a double current catheter. The danger is more theoretical than real. The os is usually patulous and the water escapes freely. The irrigation should be done with the same scrupulous care as surgical work, and should be intrusted to no other hands than the physician himself. The patient should be placed across the bed in Sims' position, with her hips resting upon Kelley's pad to protect her person and clothing. The perineum should be retracted with Sims' speculum to facilitate the easy introduction of the nozzle into the cavity of the uterus. If after the first irrigation the temperature does not rapidly subside, the curette should be used at once, fearing that blood clots or adherent placental tissue constituting a stinking mass of débris may have been retained. In future I shall pack the uterine cavity with iodoform gauze after each irrigation, after the method of my friend, Dr. William H. Polk, of New York, in treating endometritis.

This should be repeated after each irrigation until the danger of systemic intoxication has passed. The mercuric solution should not be used sufficiently long to endanger its constitutional effect. If it is imminent a 2 to 5 per cent. solution of creoline is an admirable substitute.

The patient should be sustained from the beginning with pure whisky, combined or not with milk and eggs. Quinine should also enter into our therapeutical endeavor, as the disease, as was pointed out many years ago by Fordyce Barker, is frequently associated with malaria. It should be used, also, for its antipyretic effect and its action on the vascular and nervous system. The new antipyretics should be tabooed on account of their depressing action.

I regard the treatment of puerperal septicæmia as mainly surgical,and best met by fulfilling the following indications:

1. Irrigation.
2. Curetting.
3. Drainage.
4. Abdominal section.

There are cases coming up occasionally which resist all of our therapeutic measures and yet may yield to abdominal section. In these cases the disease has passed beyond the domain of the uterine cavity ; the septic matter has become localized in the walls of the uterus, in the tubes or ovaries, or has invaded the peritoneal cavity. Any of these conditions may become of sufficient gravity to jeopardize the life of the patient.

Such cases demand section. It is not possible for us always to anticipate the pathological condition in a given case, but section enables the operator to deal with them as he finds them.

I remember seeing a case reported by Dr. Lapthorn Smith, of Montreal (*American Journal of Obstetrics and Diseases of Women*), in which he amputated the uterus and saved his patient's life. I feel confident that this method of dealing with apparently hopeless cases has a field, though a limited one, and will save many women who are doomed without it. To be effectual it must not be long delayed.

I frequently meet with good practitioners who have a superstitious dread of uterine irrigation, and who are indifferent to aseptic methods and chary of antiseptic methods. To all such I would say: There is no half-way ground.

If you believe in it you must practice it religiously. If not, then go on making premature angels of your patients in the future as in the past.

